# Integrated Methylome and Transcriptome Analyses Reveal the Molecular Mechanism by Which DNA Methylation Regulates Kenaf Flowering

**DOI:** 10.3389/fpls.2021.709030

**Published:** 2021-08-26

**Authors:** Zengqiang Li, Meiqiong Tang, Dengjie Luo, Muhammad Haneef Kashif, Shan Cao, Wenxian Zhang, Yali Hu, Zhen Huang, Jiao Yue, Ru Li, Peng Chen

**Affiliations:** ^1^Key Laboratory of Plant Genetics and Breeding, College of Agriculture, Guangxi University, Nanning, China; ^2^College of Life Science and Technology, Guangxi University, Nanning, China

**Keywords:** kenaf (*Hibiscus cannabinus* L.), DNA methylation, 5-azacytidine (5-azaC), flowering, transcriptome

## Abstract

DNA methylation regulates key biological processes in plants. In this study, kenaf seedlings were pretreated with the DNA methylation inhibitor 5-azacytidine (5-azaC) (at concentrations of 0, 100, 200, 400, and 600 μM), and the results showed that pretreatment with 200 μM 5-azaC promoted flowering most effectively. To elucidate the underlying mechanism, phytohormone, adenosine triphosphate (ATP), and starch contents were determined, and genome-wide DNA methylation and transcriptome analyses were performed on anthers pretreated with 200 μM 5-azaC (5-azaC200) or with no 5-azaC (control conditions; 5-azaC0). Biochemical analysis revealed that 5-azaC pretreatment significantly reduced indoleacetic acid (IAA) and gibberellic acid (GA) contents and significantly increased abscisic acid (ABA) and ATP contents. The starch contents significantly increased in response to 200 and 600 μM 5-azaC. Further genome-wide DNA methylation analysis revealed 451 differentially methylated genes (DMGs) with 209 up- and 242 downregulated genes. Transcriptome analysis showed 3,986 differentially expressed genes (DEGs), with 2,171 up- and 1,815 downregulated genes. Integrated genome-wide DNA methylation and transcriptome analyses revealed 72 genes that were both differentially methylated and differentially expressed. These genes, which included *ARF*s, *PP2C*, *starch synthase*, *FLC*, *PIF1*, *AGL80*, and *WRKY32*, are involved mainly in plant hormone signal transduction, starch and sucrose metabolism, and flowering regulation and may be involved in early flowering. This study serves as a reference and theoretical basis for kenaf production and provides insights into the effects of DNA methylation on plant growth and development.

## Introduction

Epigenetics refers to heritable changes in gene function with no concurrent change in DNA sequence. DNA methylation involves the addition of a methyl group to cytosine residues. This addition occurs not only on symmetric CG and CHG sequences but also on asymmetric CHH sequences (where H represents A, T, or C) (Chan et al., 2005). DNA methylation is essential for gene expression regulation, biological processes, growth and development, flowering induction, and environmental stress responses (Chan et al., 2005; He et al., 2011; Jullien et al., 2012; Shi et al., 2015; Song et al., 2020; Li et al., 2021). In higher plants, approximately 20–30% of cytosines are methylated, and a large difference in DNA methylation level exists among different plant tissues and under different conditions (He et al., 2011). Methylation in *Arabidopsis thaliana* at all sites is performed by DNA METHYLTRANSFERASE 1 (MET1), a plant-specific CHROMOMETHYLASE 3 (CMT3), and DOMAINS REARRANGED METHYLTRANSFERASEs (DRMs) (Chan et al., 2005). These DNA methylase genes regulate various stages of plant growth and development by regulating the dynamic equilibrium of genomic DNA methylation level, such as seed germination, vernalization, flowering, response to biotic and abiotic stresses, etc (Chan et al., 2005). For example, in *A. thaliana*, the global cytosine methylation level decreased in *MET1* deficient mutant, and the mutant plants had a late-flowering phenotype due to the hypomethylated of *fwa* gene, which is a well-characterized flowering time gene (Soppe et al., 2000; Kankel et al., 2003). DNA demethylation is closely linked to plant growth, development, vernalization, and flowering. Active DNA demethylation is initiated by 5′-methylcytosine DNA glycosylase/lyase enzymes, including REPRESSOR OF SILENCING 1 (ROS1), DEMETER (DME), DEMETER-LIKE 2 (DML2), and DML3 (Zhu, 2009; Zemach et al., 2013; Matzke and Mosher, 2014). 5-Azacytidine (5-azaC) is a DNA methyltransferase inhibitor and a cytosine nucleoside analog and can alter plant phenotypes, plant development, and plant methylation status (Kornicka et al., 2017). Plants subjected to 5-azaC treatment showed clear dwarfing and morphological trait abnormalities (Sano et al., 1990; Kanchanaketu and Hongtrakul, 2015). Non-vernalized *A. thaliana* and *Thlaspi arvense* plants treated with 5-azaC flowered early (Burn et al., 1993). In addition, similar results have also been reported on the effects of 5-azaC on the growth, flowering time and sexual phenotype of other higher plants (Kondo et al., 2007; Li et al., 2015; Zhou et al., 2016; Kang et al., 2019).

Kenaf (*Hibiscus cannabinus* L.) is a valuable multipurpose natural fiber crop species. Kenaf biomass is used for paper, cordage, building materials, automotive interiors, carpet backing, animal bedding, and livestock forage (Chen et al., 2021; Li et al., 2021). Kenaf is a thermophilic and short-day plant species that cannot or rarely flowers in northern China because of the prevailing long-day conditions; this is especially true for late-maturing varieties. Thus, in kenaf production, seeds must be produced in southern China and transported to northward for cultivation. At the same time, early flowering is conducive to the early harvest of kenaf, which provides time for the next crop planting. Therefore, early flowering is essential to kenaf production, especially in northern China. However, the epigenetic controls of flowering time in kenaf are unknown. Therefore, it is crucial to investigate the effects of DNA methylation on kenaf growth and development, especially the regulation of flowering time.

The objectives of this study were to explore the effects of different concentrations of 5-azaC on kenaf flowering time, physiological attributes, DNA methylation, and mRNA expression. The results could serve as a reference and theoretical basis for kenaf production and provide insights into the effect of DNA methylation on plant growth and development.

## Materials and Methods

### Plant Materials and Treatments

In this study, the kenaf (*H. cannabinus* L.) cultivar P3A was used as the plant material. Healthy P3A seeds were soaked for 10 min in 3% H_2_O_2_, washed three times in distilled water, and sown evenly in 15 plastic boxes (27 cm × 18 cm × 9 cm) that contained two layers of gauze, each plastic box was received with 300 seeds. The seeds were then treated with different concentrations of 5-azaC (0, 100, 200, 400, or 600 μM), with three replications. 5-azaC was added to each plastic box according to each corresponding concentration, and the seeds were cultivated under controlled conditions, i.e., 27°C in light for 10 h and 25°C in darkness for 14 h, with a relative humidity of 65–70% and a light intensity of 280 μmol/m^2^/s. After 5 days of 5-azaC pretreatment, 200 healthy seedlings were randomly selected from each plastic box, after which they were divided into two groups and then transplanted separately to the field at two locations: Nanning, Guangxi Province (108°E, 22°N, southern China), and Xinyang, Henan Province (114°E, 31°N, northern China). The average pH values of the topsoil in Xinyang (northern China) and Nanning (southern China) were 5.90 and 5.68, respectively, and the average organic matter contents were 18.12 and 20.81 g/kg, respectively. The average contents of total nitrogen, available phosphorus and available potassium in the two locations were 1.04 g/kg, 9.77 mg/kg, and 80.1 mg/kg, respectively (Xinyang) and 1.08 g/kg, 10.65 mg/kg, and 124.66 mg/kg, respectively (Nanning). The average temperatures of the two areas from June to September (which corresponds to the main development period of kenaf) in recent years were 23–31.3 and 25.7–33.5°C, respectively, with average precipitation amounts of 500 and 693.33 mm, respectively (Luo, 2012; Yan, 2014). The plants were cultivated and grown under natural field conditions in accordance with local management practices.

Anthers from three different plants in Nanning were harvested after plants were grown for 100 days, after which the samples were pooled together as one biological replicate. Each group was replicated three times, and a total of nine plants were sampled for physiological index measurements and DNA methylation and transcriptome analyses.

### Determination of Endogenous Hormone, Adenosine Triphosphate (ATP), and Starch Contents

The contents of indoleacetic acid (IAA), gibberellic acid (GA), and abscisic acid (ABA) in kenaf anthers pretreated with different concentrations of 5-azaC were measured via enzyme-linked immunosorbent assay (ELISA). Endogenous hormones were extracted according to the method proposed by Zur et al. (2015). Plant hormone ELISA kits purchased from Zoonbio Biotechnology Co., Ltd. (Nanjing, China) were used to measure the contents of IAA, GA, and ABA according to the kits’ protocols (ELISA kits BH100, BH101, and BH102). Similarly, the adenosine triphosphate (ATP), starch and soluble sugar contents were determined via kits (Solarbio Science & Technology, Beijing, China, kits BC0300, BC0700, and BC0030).

### MethylRAD Sequencing and Data Analysis

Genomic DNA was extracted from anthers via a DNA Secure Plant Kit (Cat. #DP320-03, TIANGEN, Beijing, China) according to the manufacturer’s protocol. The DNA was then assessed via electrophoresis on agarose gels, and the quality and concentration of the DNA were determined via a NanoDrop 2000 spectrophotometer. Approximately 150–200 ng of DNA was used to construct MethylRAD libraries following a previous protocol (Wang et al., 2015). Briefly, total DNA was digested via the methylation-dependent restriction enzyme *FspE*I (NEB, United States). *FspE*I can recognize 5-methylcytosine (5-^m^C) and 5-hydroxymethylcytosine (5-h^m^C) within C^m^CGG and ^m^CHG sites and produce a double-stranded DNA break at a fixed distance from the modified cytosine. Accordingly, symmetrical DNA methylated sites were bidirectionally cleaved by *FspE*I to generate 32-base-long fragments. Two adapters were then added to the digested DNA by T4 DNA ligase (NEB, United States), after which the ligation products were amplified via specific primers. The target DNA fragments were further purified and subjected to sequencing on an Illumina HiSeq X^TM^ Ten platform.

The raw reads were first subjected to quality filtering and adapter trimming. Reads with more than 8% ambiguous bases (N), reads of poor quality (15% nucleotide positions with a Phred quality <30) or reads without enzyme restriction sites were removed by a custom Perl script. The reads with enzyme sites (enzyme reads) were subsequently aligned against the kenaf genome^1^ by Bowtie 2 (version 2.3.4.1), with the default parameters. The distribution of the methylated cytosine sites among kenaf chromosomes and different functional elements of the genome were evaluated. The relative expression level of each methylated site (CpG) was determined by a normalized read depth—reads per million (RPM; equal to read coverage per site/high-quality reads per library × 1,000,000). The changes in methylation level were assessed on the basis of the sequencing depth of each site in the relative quantitative methylation results by the R package DESeq. The different *p*-values and fold-changes (FCs) among different sites were calculated according to the sequencing data (*P* < 0.05, log_2_|*FC*| > 1). Differentially methylated genes (DMGs) were analyzed by Gene Ontology (GO) and Kyoto Encyclopedia of Genes and Genomes (KEGG) functional enrichment analysis.

### Transcriptome Sequencing and Data Analysis

The total RNA from the anthers was extracted according to a modified cetyltrimethylammonium bromide (CTAB) RNA extraction protocol. The RNA purity and quantification were evaluated with a NanoDrop 2000 spectrophotometer (Thermo Fisher Scientific, Waltham, MA, United States), and the RNA integrity was assessed with an Agilent 2100 Bioanalyzer (Agilent Technologies, Santa Clara, CA, United States) (Chen et al., 2021). cDNA libraries were then constructed with a TruSeq Stranded mRNA LT Sample Prep Kit (Illumina, San Diego, CA, United States) according to the manufacturer’s instructions.

The libraries were sequenced on an Illumina HiSeq X^TM^ Ten platform, and 150-bp paired-end reads were generated. The raw data in fastq format were first processed with Trimmomatic, and raw reads for each sample were generated. After reads containing adapters, reads containing poly-N bases and reads of low quality were removed from the raw data, clean reads were obtained for subsequent analyses. The clean reads were then mapped to the kenaf genome (see text footnote 1) by HISAT2.

The fragments per kilobase of transcript per million mapped reads (FPKM) value of each gene was calculated with Cufflinks, and the read counts were obtained by the use of htseq-count. Differential gene expression was analyzed via the DESeq R package; *P* < 0.05 and *FC* > 2 or *FC* < 0.5 were set as the thresholds for significantly different expression. Hierarchical cluster analysis of differentially expressed genes (DEGs) was performed to explore gene expression patterns. GO functional enrichment and KEGG pathway enrichment analysis of the DEGs was performed with R on the basis of a hypergeometric distribution. Gene structure extension and novel transcript identification were performed by comparing the reference genome and the known annotated genes via Cuffcompare software.

ASprofile was used to analyze the alternative splicing of differentially regulated transcript isoforms or exons. Single-nucleotide polymorphisms (SNPs) and insertions and deletions (INDELs) were called using SAMtools and BCF tools. The details are shown on the SAMtools webpage.^2^ SnpEff was then used to annotate and predict the effects of variants on genes (such as amino acid changes).

### qRT-PCR Analysis

Kenaf anther RNA was extracted and reverse transcribed into cDNA, which was used as a template for qRT-PCR. qRT-PCR was performed using ChamQ Universal SYBR qPCR Master Mix (Vazyme Biotech Co., Ltd.) on a Bio-Rad CFX96 instrument (Bio-Rad Laboratories). Each 20 μL reaction mixture was composed of 1.0 μL of cDNA, 0.4 μL of primers (Supplementary Table 1), 10 μL of ChamQ Universal SYBR qPCR Master Mix and 8.2 μL of nuclease-free water. The amplification program was as follows: 94°C for 3 min, followed by 40 cycles of 94°C for 10 s and 60°C for 30 s. All the reactions were performed in triplicate for each sample. According to the previous screening results of internal reference genes in our laboratory, *Actin 3*, *Histone 3*, and *18 SrRNA* were used as endogenous reference genes for normalizing gene expression, and the relative gene expression was calculated via the 2^–ΔΔCT^ method in this study (Chen et al., 2014; Zhou et al., 2017).

### Statistical Analysis

The data concerning the contents of IAA, GA, ABA, ATP, and starch are presented as the means ± standard errors (SEs) of three replicates. One-way variance analysis (ANOVA) was performed via SPSS Version 16 (SPSS, Inc., Chicago, IL) software. The means were analyzed via the least significant difference (LSD) test at *P* ≤ 0.05 for statistical significance.

## Results

### Effects of 5-azaC on Morphological Indexes

**FIGURE 1:**
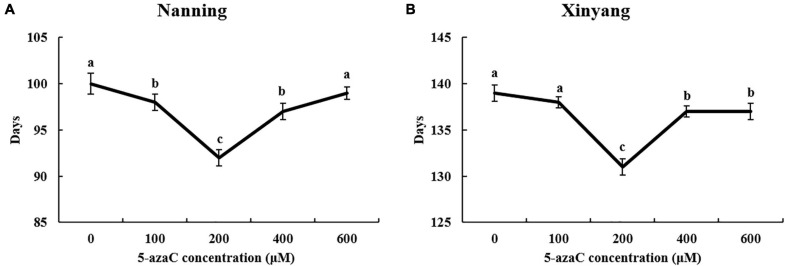
Kenaf flowering time under different concentrations of 5-azaC pretreatment. **(A)** Days required for the early blooming stage at Nanning. **(B)** Days required for the early blooming stage at Xinyang.

The results showed that 5-azaC pretreatment could shift the flowering time of kenaf, and the effects of 5-azaC on flowering were closely related to the concentration used. Low-concentration (100 μM) and high-concentration (600 μM) pretreatment had no significant effects on flowering. Moderate concentrations (200 and 400 μM) resulted in early flowering, and the effects were significantly more obvious in Xinyang (northern China). In Nanning and Xinyang, pretreatment with 200 and 400 μM 5-azaC advanced start of the early blooming stage by 5–7 and 2–4 days (Figures 1A,B) and the start of the blossom period by 5–7 and 2–3 days, respectively. The early flowering caused by 5-azaC is very important with respect to kenaf production, especially in northern China.

### Effects of 5-azaC on Physiological Indexes

**FIGURE 2:**
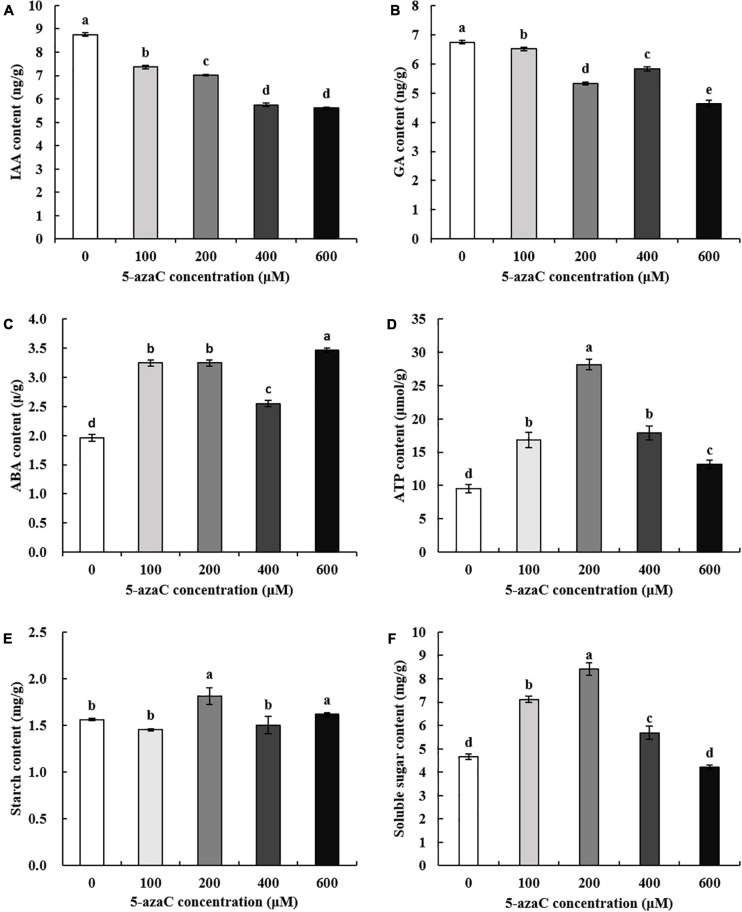
Response of phytohormones to different concentrations of 5-azaC. **(A)** IAA content, **(B)** GA content, **(C)** ABA content, **(D)** ATP content, **(E)** starch content, **(F)** Soluble sugar content. The different lowercase letters indicate significant differences at the *P* < 0.05 level.

5-azaC pretreatment significantly reduced the contents of IAA and GA in kenaf anthers (Figures 2A,B) and significantly increased the ABA contents (Figure 2C). In addition, the ATP content significantly increased in 5-azaC-pretreated kenaf anthers (Figure 2D). The starch content significantly increased under pretreatment with 200 and 600 μM 5-azaC (Figure 2E), and the soluble sugar content significantly increased under pretreatment with 100, 200, and 600 μM 5-azaC (Figure 2F). In particular, the ATP, starch and soluble sugar contents in kenaf anthers under 5-azaC200 significantly increased by 194.45%, 16.21%, and 80.69%, respectively, compared with the control levels. On the basis of these results, kenaf anthers in the 5-azaC200 and 5-azaC0 groups were chosen for subsequent experiments.

### DNA MethylRAD Sequencing and DNA Methylation Levels

To obtain an overview of kenaf cytosine methylation patterns, three libraries were constructed from control (5-azaC0) anthers and those pretreated with 5-azaC (5-azaC200) separately and then sequenced on the MethylRAD sequencing platform. On average, 29.02 million raw reads were obtained for the 6 libraries. After the labels without the expected restriction sites were filtered and removed, the MethylRAD sequencing data (enzyme reads) comprised 5.18, 4.78, 4.96, 4.74, 5.00, and 4.90 million reads for 5-azaC0 and 5-azaC200. Finally, 0.79, 0.74, 0.74, 0.72, 0.77, and 0.69 million mapping reads were generated (enzyme reads with unique alignment locations on the reference sequence). The enzyme reads were submitted to the Sequence Read Archive (SRA)^3^ of the NCBI (accession number: SRR11743644-SRR11743649).

On average, 121,596 CCGG and 58,958 CCWGG DNA methylation sites with average depths of 4.26 and 4.29, respectively, were found for 5-azaC0, and on average, 118,747 CCGG and 57,674 CCWGG DNA methylation sites with average depths of 4.19 and 4.21, respectively, were found for 5-azaC200. These results showed that the level of DNA methylation at the CCGG sites was greater than that at the CCWGG sites in kenaf anthers, and the level of DNA methylation decreased by 2.29% after treatment with 200 μM 5-azaC compared with the control (Table 1).

**TABLE 1:** Summary of methylation site coverage.

Sample	CCGG		CCWGG	
		
	Site number	Depth	Site number	Depth
5-azaC0-1	121,361	4.47	58,933	4.47
5-azaC0-2	122,972	4.13	58,878	4.11
5-azaC0-3	120,454	4.17	59,062	4.28
5-azaC200-1	120,275	4.11	57,960	4.13
5-azaC200-2	121,015	4.47	57,616	4.20
5-azaC200-3	114,950	3.99	57,445	4.31

### Methylation Site Distribution Within the Kenaf Genome

**FIGURE 3:**
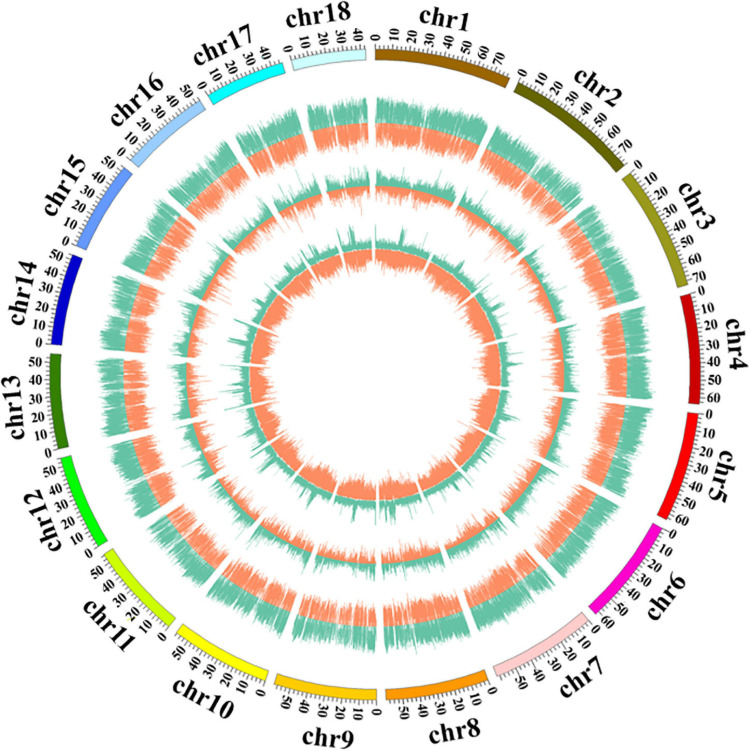
Distribution of DNA methylation sites across kenaf chromosomes. From outside to inside, the chromosome coordinates, read depth, number of methylation sites in each window, and number of enzyme restriction sites in each window were determined. The different color represent different types of methylation sites: green represents the CCGG site (the greater the value is, the farther out the line extends), and orange represents the CCWGG site (the greater the value is, the farther in the line extends).

This study is the first to systematically analyze the distribution of DNA methylation sites on each chromosome of kenaf. The distributions of CCGG methylation sites and CCWGG methylation sites across the chromosomes of kenaf were consistent, and most methylation sites were present on the chromosome 12, followed by chromosome 3. Chromosome 11 had the fewest methylation sites, followed by the chromosome 2. A map of the distribution of DNA methylation sites on each chromosome of kenaf is shown in Figure 3.

### Distribution of Methylation Sites Within Different Functional Elements and Gene Regions

**FIGURE 4:**
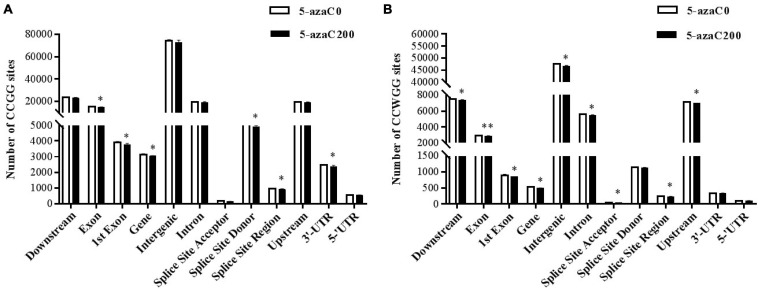
Distribution of methylation sites within functional elements under 5-azaC pretreatment. **(A)** Distribution of CCGG methylation sites within functional elements. **(B)** Distribution of CCWGG methylation sites within functional elements. A single asterisk indicates a significant difference at the *P* < 0.05 level, and a double asterisk indicates a significant difference at the *P* < 0.01 level.

The number and distribution of methylation sites within different gene elements of the kenaf genome were calculated. The results showed that the CCGG and CCWGG methylation sites lied mostly within intergenic regions, followed by downstream regions, upstream regions, introns and exon (Figure 4 and Supplementary Table 2). The fewest sites lied in splice site acceptor regions, followed by the 5′-UTRs, splice site regions and 3′-UTRs. There were significantly fewer numbers of CCGG and CCWGG methylation sites within each functional elements in 5-azaC200 than in 5-azaC0.

**FIGURE 5:**
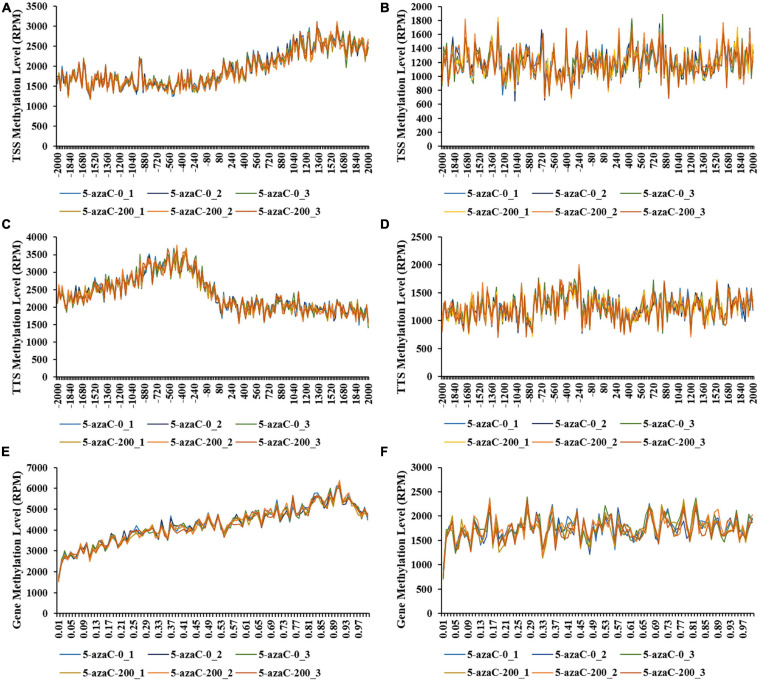
DNA methylation levels in different gene regions under 5-azaC pretreatment. **(A,B)** indicate the DNA methylation levels at the CCGG and CWGG sites of the transcription start site (TSS) region, respectively. **(C,D)** indicate the DNA methylation levels at the CCGG and CCWGG sites of the transcription termination site (TTS) region, respectively. **(E,F)** indicate the DNA methylation levels at the CCGG and CCWGG sites of the gene coding region, respectively.

The distribution of methylation sites was analyzed within 2-kb regions upstream and downstream of the transcription start site (TSS; Figures 5A,B), transcription termination site (TTS; Figures 5C,D) and gene coding regions (Figures 5E,F). The results showed that the gene coding regions had the greatest numbers among all the samples, and the number of CCGG methylation sites was significantly lower in the 5-azaC200 group than in the 5-azaC0 group (Figure 5E).

### Differentially Methylated Sites (DMSs) and Genes (DMGs)

In total, 862 CCGG-site DMSs and 323 CCWGG-site DMSs were identified in the 5-azaC0 and 5-azaC200 samples (Supplementary Table 3). There were 402 up- and 460 downregulated CCGG-site DMSs and 150 up- and 173 downregulated CCWGG-site DMSs in the 5-azaC200 samples compared with the control samples.

In addition, the methylation levels of DMGs were calculated (Supplementary Table 3). Among 1,185 DMSs, 451 DMGs were identified, including 384 CCGG-site DMGs and 67 CCWGG-site DMGs. Among them, 181 and 203 were up- and downregulated CCGG-site DMGs, and 28 and 39 were up- and downregulated CCWGG-site DMGs, respectively.

### GO and KEGG Functional Enrichment Analysis of DMGs

**FIGURE 6:**
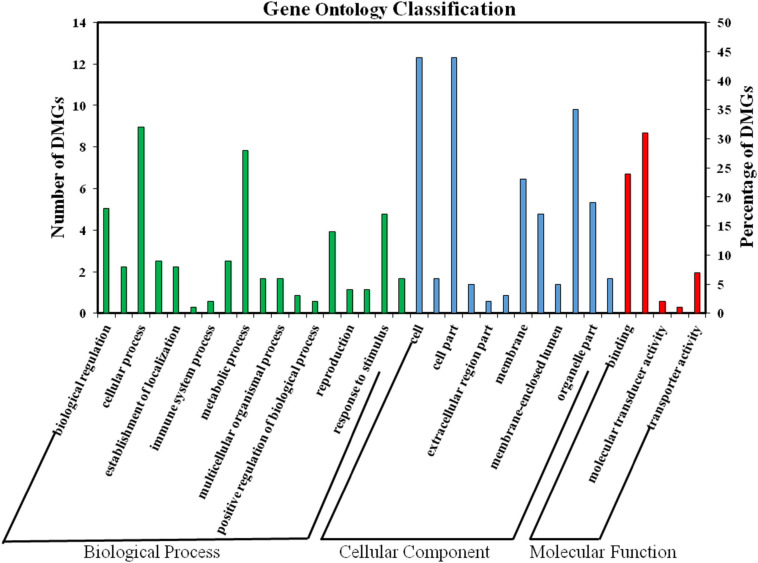
GO enrichment of DMGs.

To further characterize the changes in DNA methylation levels in these two samples, we performed GO and KEGG enrichment analysis of the DMGs (Supplementary Table 4). As shown in Figure 6, a total of 35 highly enriched GO terms were assigned. The main terms in the biological process category included “cellular process”, “metabolic process”, “biological regulation”, and “reproduction”. The main cellular components included the “cell”, “organelle”, “membrane”, and “symplast” and the main molecular function terms were “catalytic activity”, “binding”, and “transporter activity”.

**FIGURE 7:**
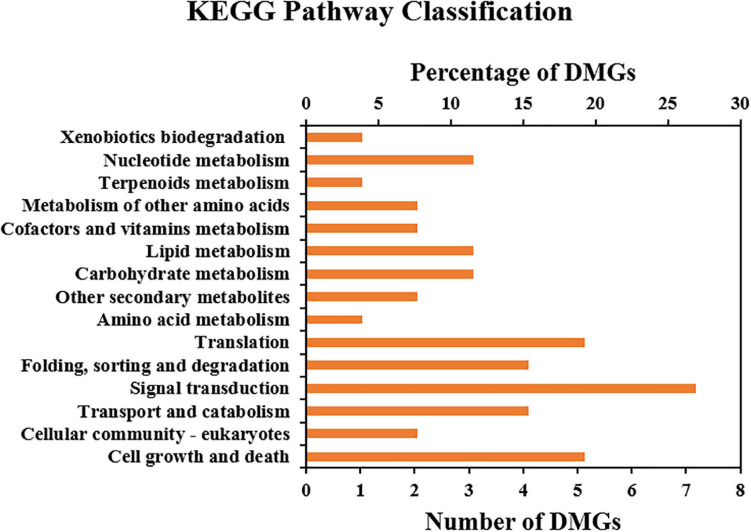
KEGG enrichment of DMGs.

KEGG enrichment analysis indicated that 31 DMGs (13 and 18 of which were up- and downregulated, respectively, under 5-azaC200) were highly enriched in 16 pathways. The main significant pathways included “signal transduction”, “translation”, “carbohydrate metabolism”, “lipid metabolism”, “nucleotide metabolism”, “biosynthesis of other secondary metabolites”, “amino acid metabolism”, “cell growth and death”, “transport and catabolism”, and so on. These are essential for plant growth and development and are suggested to be involved in the regulation of flowering (Figure 7).

### Transcriptome Assembly and Functional Annotation

To assess the gene expression profile during flowering, RNA was extracted from anthers (three replicates) in each of the experimental groups (5-azaC0 and 5-azaC200) and then sequenced on the Illumina HiSeq X Ten^TM^ platform. An average of 49.42 million raw reads were obtained for the six libraries. After filtering was performed, 48.07 million clean reads were obtained on average in each of the 6 libraries, the average GC content was 46.16%, and their Q30 values were greater than 94% (Supplementary Table 5). The complete set of clean reads of these libraries have been deposited in the SRA (see text footnote 3) of the NCBI under the accession numbers SRR10912354-SRR10912359. The clean reads were compared with the kenaf reference genome sequence, and an average of 45.02 million unique mapped reads were obtained, with an average comparison rate of 93.66% (Supplementary Table 6).

Additionally, we confirmed the reliability of the samples via correlation analyses based on FPKM values. The high similarity (*r* > 0.99) between the three biological replicates of 5-azaC0 and 5-azaC200 indicated that the RNA sequencing results were reliable.

### Analysis of Differentially Expressed Genes (DEGs)

Differentially expressed genes (DEGs) between 5-azaC200 and 5-azaC0 were identified on the basis of the following threshold values: log_2_|*FC*| > 1 and *P* < 0.05. A total of 3,986 DEGs, 2,171 (54.47%) and 1,815 (45.53%) of which were up- and downregulated, respectively, were identified from the comparison of 5-azaC200 and 5-azaC0 (Supplementary Table 7). These data indicate that most of the expression of most DEGs increased under 5-azaC200 by demethylation.

**FIGURE 8:**
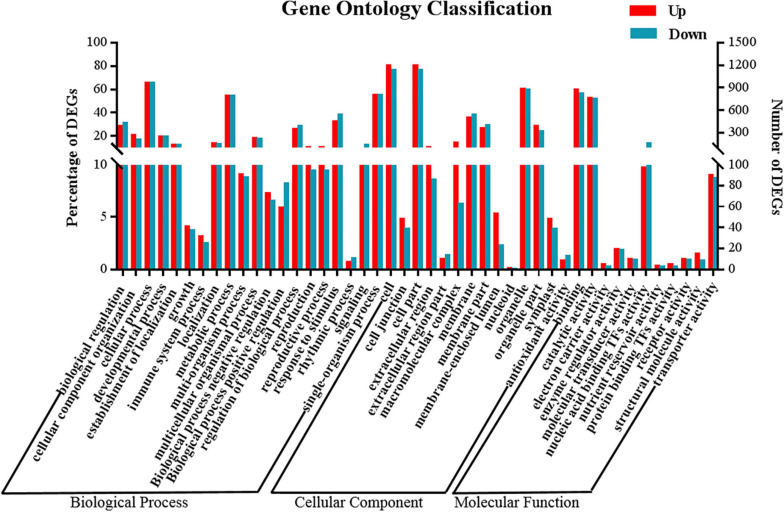
Distribution of up- and downregulated DEGs at GO level 2.

GO and KEGG enrichment analysis was performed to better understand the functions of the DEGs whose expression was induced in response to 5-azaC pretreatment in terms of the regulation of kenaf flowering time (Supplementary Table 8). GO enrichment analysis (Figure 8) revealed that 2,531 DEGs (of which 1,346 and 1,185 were up- and downregulated, respectively, under 5-azaC200) were highly enriched in 629 GO terms, 391 of whose genes were upregulated and 238 of whose genes were downregulated under 5-azaC200. Most of the terms were similar to those identified in the analysis of DMGs. The GO terms associated with upregulated DEGs in the biological process category included mainly “cellular process”, “developmental process”, “metabolic process”, “reproductive process”, and “multicellular organismal process”. The GO terms associated with upregulated DEGs in the cellular component category included mainly “cell”, “organelle”, “macromolecular complex” and “cell junction”, and the GO terms associated with upregulated DEGs in the molecular function category included mainly “binding”, “catalytic activity”, and “structural molecule activity”. The GO terms associated with downregulated DEGs included mainly “signaling”, “response to stimulus”, “membrane”, “antioxidant activity”, and “nucleic acid binding transcription factor activity”.

**FIGURE 9:**
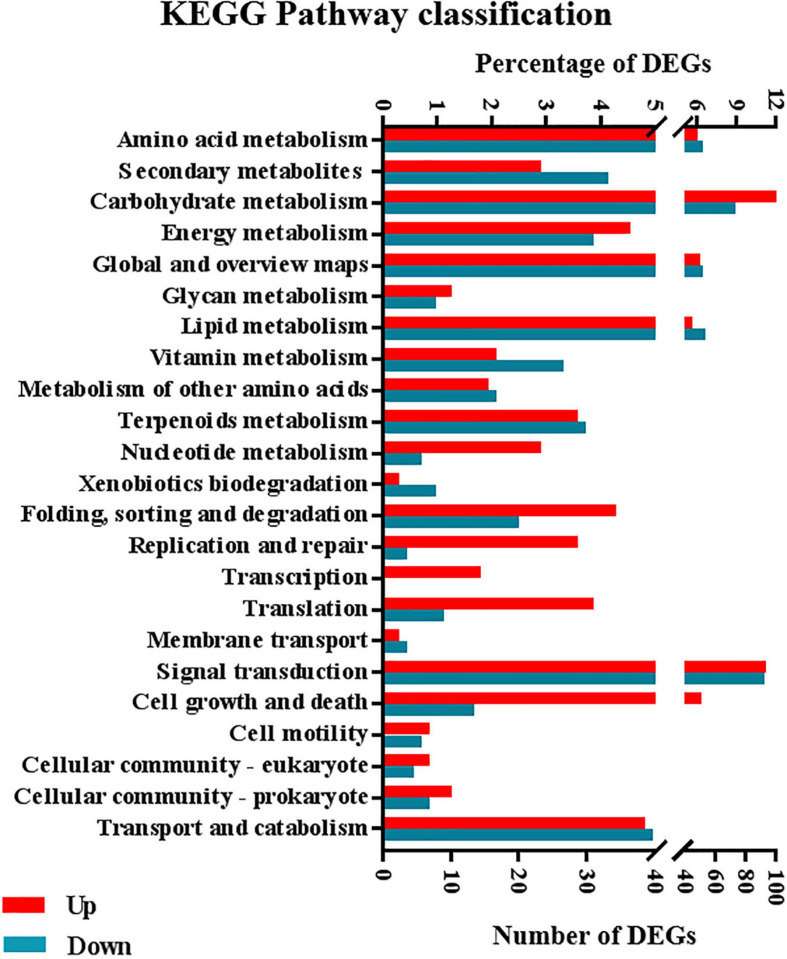
Distribution of up- and downregulated DEGs at KEGG level 2.

KEGG enrichment analysis revealed that 731 DEGs, including 405 and 326 of which were up- and downregulated, respectively, under 5-azaC200, were enriched in 23 pathways. Remarkably, the upregulated DEGs were enriched mainly in “carbohydrate metabolism”; “energy metabolism”; “nucleotide metabolism”; “cell growth and death”; “folding, sorting, and degradation”; “translation”; “transcription”; and “replication and repair”. The downregulated DEGs were enriched mainly in “lipid metabolism”, “biosynthesis of other secondary metabolites”, “metabolism of cofactors and vitamins”, and “xenobiotic biodegradation and metabolism” (Figure 9). These pathways associated with the identified DEGs might provide a basis for further analysis of genes involved in regulating kenaf flowering time.

In addition, 19,256 transcription factors were distributed among 58 families. A total of 3,263 differentially expressed transcription factors (DTFs) were distributed across 57 families, including the bHLH, MYB, MADS, C2H2, WRKY, CO-like, and LFY families, whose members are closely associated with flowering (Table 2). Among those, at least 1,772 DTFs were upregulated and 1,491 downregulated under 5-azaC200.

**TABLE 2:** Gene family distribution of different transcription factors (DTFs).

TF family	The number of DTFs	Up-	Downregulated	TF family	The number of DTFs	Up-	Downregulated
bHLH	303	171	132	GeBP	31	17	14
ERF	246	104	142	DBB	30	19	11
NAC	238	119	119	Dof	29	17	12
MYB-related	181	94	87	TALE	29	8	21
WRKY	181	113	68	GATA	28	16	12
C2H2	161	85	76	S1Fa-like	28	25	3
MYB	118	61	57	Nin-like	26	15	11
B3	108	58	50	CPP	25	19	6
C3H	101	61	40	CO-like	22	13	9
bZIP	97	53	44	E2F/DP	21	12	9
G2-like	93	44	49	ARR-B	20	13	7
FAR1	91	52	39	GRF	20	12	8
M-type-MADS	89	51	38	BBR-BPC	17	6	11
LBD	85	40	45	HB-PHD	14	9	5
Trihelix	84	50	34	YABBY	11	4	7
GRAS	76	45	31	AP2	9	6	3
HD-ZIP	69	45	24	EIL	8	6	2
ARF	63	47	16	WOX	8	3	5
HSF	50	29	21	ZF-HD	8	2	6
TCP	47	21	26	LSD	7	3	4
MIKC-MADS	45	28	17	RAV	7	5	2
NF-YA	43	22	21	HRT-like	5	2	3
NF-YC	43	19	24	Whirly	4	3	1
BES1	42	22	20	LFY	3	3	0
HB-other	42	24	18	NF-X1	3	2	1
SBP	42	23	19	NZZ/SPL	2	0	2
NF-YB	40	23	17	SRS	1	0	1
STAT	36	14	22	VOZ	1	1	0
CAMTA	32	13	19	SAP	0	0	0

### Integrated Analysis of DMGs and DEGs

**FIGURE 10:**
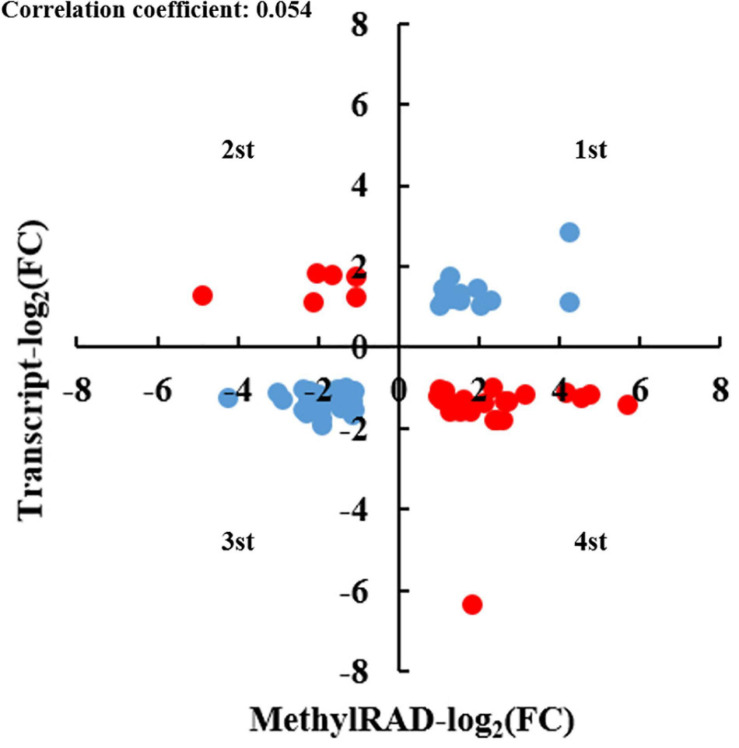
Quadrant chart analysis of the relationship between DMG expression and DEG expression.

To better understand the underlying mechanism through which DNA methylation regulates kenaf flowering, an integrated analysis of anthers in the 5-azaC0 and 5-azaC200 groups was conducted on the basis of the methylome and transcriptome data. By comparing 451 DMGs and 3,986 DEGs, we identified a total of 72 genes that represented both DMGs and DEGs (Supplementary Table 9). To more accurately assess the potential correlation between DNA methylation and gene expression, a four-quadrant graph was constructed. As shown in Figure 10, 14, 6, 27, and 25 genes were located in the first to fourth quadrants, respectively. The location of genes such as *auxin response factor 18 (ARF18)*, *starch synthase*, *glutaredoxin family protein*, *serine/threonine-protein kinase CDL1*, *E3 ubiquitin-protein ligase LIN-1*, *ABC transporter G family member 25*, *indole-3-acetaldehyde oxidase*, *phosphatase 2C family protein*, *cytokinin dehydrogenase 7*, *cytochrome P450 83B1*, and *cytochrome P450 CYP72A219* in the first and third quadrants implied that DNA methylation may positively regulate gene expression. Conversely, the location of genes in the second and fourth quadrants implied that DNA methylation may negatively regulate gene expression. Examples of these genes include *cytochrome P450*, *ethylene-responsive element-binding protein*, *mitogen-activated protein kinase*, *plastid developmental protein*, *cellulose synthase-like protein E1*, *ABC transporter G family*, *histone-lysine N-methyltransferase*, and *lipid-transfer protein*. The reason about the appearance of two opposing regulatory modes, since the genomic location, underlying DNA sequence, and the type of DNA methylation is often reflective of its expression status (Bewick and Schmitz, 2017). We subsequently mapped these genes to GO and KEGG pathways (Supplementary Table 10). The most represented GO terms included “starch biosynthetic process”, “regulation of transcription”, “nucleus”, “chloroplast”, “cytoplasm”, “amyloplast”, “glycogen (starch) synthase activity”, “O-acetyltransferase activity”, and “DNA binding”. Among the KEGG pathways, “plant hormone signal transduction”, “carbon metabolism”, “fatty acid metabolism”, and “peroxisome” were the four most represented pathways.

### Expression Verification via qRT-PCR

To verify the accuracy of our transcriptome sequencing, 24 candidate DEGs were selected and analyzed via qRT-PCR. As shown in Table 3, the expression patterns of most of the genes showed general agreement with the transcriptome sequencing results. Only the *PPR-containing protein* genes exhibited inconsistent patterns. Taken together, these results suggested that the results of the DEG analysis were reliable.

**TABLE 3:** qRT-PCR validation of the RNA-seq results.

Gene name	Gene annotation	Fold change (5-azaC200 vs 5-azaC0)		
		
		Methylation	Transcriptome	qRT-PCR
*rps5*	40S ribosomal protein S5	3.07	2.47	2.3
*ABCG25*	ABC transporter G family member 25-like	2.24	0.39	0.59
*ABCG26*	ABC transporter G family member 26	2.40	23.54	24.46
*ARF11*	auxin response factor 11-like	0.30	2.39	3.65
β*-1,2XylT*	beta-(1,2)-xylosyltransferase-like	2.40	2.10	2.94
*CAT6*	cationic amino acid transporter 6, chloroplastic-like	3.12	0.20	0.28
*CSLE1*	cellulose synthase-like protein E1	3.53	5.51	2.19
*CKX7*	cytokinin dehydrogenase 7-like	2.40	0.28	0.31
*LIN*	putative E3 ubiquitin-protein ligase LIN-1	0.50	4.12	2.83
*Egs*	endoglucanase-like	0.30	0.49	0.60
*CSLA2*	glucomannan 4-beta-mannosyltransferase 2-like	0.50	2.06	3.59
*IAAldO*	indole-3-acetaldehyde oxidase-like isoform X1	2.05	0.35	0.41
*IRX9*	beta-1,4-xylosyltransferase IRX9	2.12	0.22	0.10
*laccase-22*	laccase-22-like	2.40	0.05	0.06
*GSO1*	LRR receptor-like serine/threonine-protein kinase GSO1	2.26	0.25	0.12
*MAPK7*	mitogen-activated protein kinase 7-like	2.76	3.52	2.78
*P450-83B1*	cytochrome P450 83B1-like	3.17	0.46	0.80
*CYP72A 219*	cytochrome P450 CYP72A219-like	0.47	0.23	0.13
*PP2C46*	protein phosphatase 2C 46	2.40	0.31	0.29
*PP2C60*	protein phosphatase 2C 60	2.02	0.40	0.56
*PPR*	Pentatricopeptide (PPR) repeat-containing protein	2.57	6.37	0.30
*SLC2*	sodium/hydrogen exchanger 2-like	0.30	0.32	0.41
*SIK-CDL1*	serine/threonine-protein kinase CDL1	3.08	2.95	3.07
*ZC3H6*	zinc finger CCCH domain-containing protein 6-like	3.38	0.26	0.64

*The correlation of Methylation and Transcriptome is 0.11, and the correlation of Transcriptome and qRT-PCR is 0.95.*

## Discussion

### DNA Methylation Regulates Flowering Through Its Involvement in Carbon Metabolism

Carbohydrates are the main product of plant photosynthesis and the main source of energy for maintaining plant growth and development. For instance, sucrose and starch play important roles in plant growth and development, seed germination, flowering, and the accumulation of stored substances (Du et al., 2020). *Starch synthase* is a key enzyme involved in starch biosynthesis. The main function of *alpha-amylase* is to hydrolyze starch to form glucose. *Sucrose-phosphate synthase* (*SPS*) is the key enzyme of sucrose synthesis, which is strongly linked to plant growth, photosynthesis characteristics, and fruit ripening (Ma et al., 2020). *6-Phosphofructokinase* (*PFK*) is a key catalytic enzyme involved in fructose phosphorylation and plays an important role in fructose metabolism (Geng et al., 2018). *PFK* is also a key enzyme in the glycolysis pathway and a rate-limiting enzyme. *FRK* can shorten the time to flowering and increase the number of flowers (Odanaka et al., 2002). The expression levels of these genes, and the contents of starch and soluble sugar were substantially increased in response to 5-azaC200. We speculated that the expression of most key genes in carbohydrates metabolic pathways resulted in a more sufficient energy supply in 5-azaC-pretreated kenaf anthers, which played an important role in early flowering.

### Hormone Related Genes Were Involved in Kenaf Flowering

Plant flowering was completed under a complex flowering regulatory network formed by a variety of exogenous and endogenous signals. As the most important participants of endogenous signals, plant hormones play an important role in the flowering process. Gibberllins (GA), the main signal factors in gibberellin pathway, plays a key role in the flower-forming process, which promotes the flowering of plants mainly by regulating *GID1* (GA insensitive dwarf 1) and DELLA protein (Ross et al., 2011; Cheng et al., 2015; Bao et al., 2020). The inhibition and promotion of flowering by auxin (IAA) vary from plant to plant. More plants are inhibited than are promoted. The level of endogenous IAA in many plants is negatively correlated with flower organ development, and exogenous application of different concentrations of IAA will affect the normal development of flower organs (Wang et al., 2019; Zou et al., 2020). Whether ABA signals participate in flower bud differentiation is still controversial, and many reports are opposite to each other (Shu et al., 2016). In this study, we found that the contents of IAA and GA decreased significantly under pretreatment with 5-azaC, while the ABA contents increased significantly (Figure 2). This suggested that the changes of IAA, GA, and ABA contents were associated with the changes of flowering time.

Increasing numbers of studies suggested that there is a close relationship between epigenetic regulation and plant hormone signaling. Phytohormone play an important role in chromatin compaction mediated by DNA methylation and histone modifications. At the same time, DNA methylation also regulates plant hormone signal transduction (Zhu, 2010; Yamamuro et al., 2016; Campos-Rivero et al., 2017). Treating *Arabidopsis* and *Thlaspi* with 5-azaC can bloomearly. Thus, the authors proposed that 5-azaC promotes flowering by demethylation of the flower-related gene activate the transcription, and they further suggested the transcription of kaurenoic acid hydroxylase, a key enzyme is required for GA biosynthesis (Burn et al., 1993). Here, combined methylome and transcriptome analysis showed that a total of 75 DEGs were enriched in plant hormone signal transduction. Among them, 44 were downregulated, including *PP2C*, *EIN3*, *SNRK2*, *gibberellin receptor* (*GID1*), *ethylene receptor* (*ETR*), and *phytochrome interacting factor 4* (*pif4*). Meanwhile, the remaining 31 genes were upregulated, including *auxin response factor*s (*ARF*s), those encoding DELLA proteins, ABA receptor *PYR/PYL* family members, *brassinosteroid* (*BR*) *signaling kinase*, etc. Interestingly, genes such as *PP2C*, *EIN3*, *ARF*s, and *SNPK2* were not only differentially expressed but also differently methylated.

ABA can promote flowering by increasing the expression of *GIGANTEA* (*GI*), the flowering site T gene (*FT*) and *TWIN SISTER OF FT* (*TSF*) (Riboni et al., 2016). *PP2C*, a negative regulator, plays an essential role in ABA signal transduction (Rolland et al., 2006). In the present study, the DNA methylation and gene expression levels of *PP2C* were significantly reduced. Therefore, it was suggested that the ABA content was closely related to the level of methylation and expression of *PP2C*, which may play an essential role in promoting early flowering in kenaf. *ARF* family genes are the key regulators of auxin signaling and play an important role in auxin-mediated transcriptional regulation (Zhao, 2010). The level of methylation within *ARF11* was relatively low, but *ARF11* gene expression substantially increased. These results suggested that DNA methylation played an important role in early flowering by regulating the IAA content and related gene expression. GID1 and DELLA proteins participate in gibberellin signal transduction in plants. When the expression of GID1 proteins decreases, the gibberellin signaling pathway becomes blocked, causing DELLA proteins to accumulate in plants, thus inhibiting plant growth. DELLA proteins are the key limiting factor in the GA signaling pathway, which is involved in limiting plant growth and the regulation of flowering (Hirano et al., 2008; Jiang and Yu, 2009; Zhao, 2010). The accumulation of DELLA proteins promotes the expression of the flower development-related gene *API*, thus promoting flower development (Yamauchi et al., 2004). Our results are consistent with those of previous reports, indicating that early flowering may be related to decreased *GIDI* and *PP2C* expression, increased accumulation of DELLA proteins, and increased *PYR/PYL* expression.

### DNA Methylation Regulates the Gene Expression of DTFs Involved in the Regulation of Flowering

Transcription factors compose a class of DNA-binding proteins that regulate gene expression by binding to *cis*-acting elements in the promoter region of a gene or functional regions of other transcription factors. TFs play a central role in regulating plant growth and development, defense responses, and biotic and abiotic stress responses (Rushton et al., 2010; Agarwal et al., 2011). In the present study, many DTFs were closely related to plant flowering regulation. For example, there were 303, 299, 238, 181, 161, 134, 28, and 22 bHLH, MYB, NAC, WRKY, C2H2, MADS, GATA, and CO-like transcription factors, respectively; in each class of DTFs, most of the genes were upregulated, with 171, 155, 119, 113, 85, 79, 16, and 13 such genes, respectively.

MADS-box transcription factors have been shown to play a vital role in the regulation of floral organ development and flowering time (Theißen et al., 2016). With the exception of *AP2*, all the genes involved in the ABCDE model of floral organ development belong to different functional categories of MADS-box genes (Zhang et al., 2013). In terms of the regulation of flowering time, MADS proteins can act as either inhibitors or activators. In *Arabidopsis*, the main inhibitory factors include *FLOWERING LOCUS C* (*FLC*), *FLOWERING LOCUS M* (*FLM*), *MADS AFFECTING FLOWERING 1* (*MAF1*), MAF2 to MAF5 FLC-like proteins, *AGAMOUS-LIKE 15* (*AGL15*), and *AGL18*. Factors that promote flowering include *SOC1* and *AGL24* (Bäurle and Dean, 2006). The expression of the *AGL15* and *AGL18* genes in the anthers in the 5-azaC pretreatment group was only one-third of that in the control group. *SVP* can inhibit the late-flowering phenotype caused by *FLM* overexpression (Scortecci et al., 2003). In the present study, the expression of *SVP* significantly increased under 5-azaC200 (by 4.57-fold). In addition, the genes encoding some DTFs such as *MADS-box protein A*, *AGL62* and *AGL80* were also differentially methylated. Thus, the level of DNA methylation might affect gene expression and play an important role in early flowering.

WRKY transcription factors are important factors that regulate different functions in higher plants (Bakshi and Oelmller, 2014). Specifically, WRKY transcription factors regulate plant flowering. For example, overexpression of the *MiWRKY12* gene can promote the expression of flowering-related genes such as *CONSTANS* (*CO*), thus leading to the early *Miscanthus* flowering (Yu et al., 2013). Similarly, *GsWRKY2*0-overexpressing plants bloomed earlier than did wild-type *Arabidopsis* plants both either long and short days, and *GsWRKY20*, a member of the WRKY family in *Glycine soja*, may regulate the flowering-related genes *CO*, *FLC*, *FT*, and *SOC1* and the flower meristem-related genes *AP1*, *AP3*, and *agamous* (*AG*) to cause early flowering of soybean (Luo et al., 2013). Overexpression of *GmWRKY58* and *GmWRKY76* can promote flowering and stem growth (Yang et al., 2016), whereas *AtWRKY71* can promote *Arabidopsis* flowering (Yu et al., 2013). The transcription factors encoded by *OsWRKY24* and *OsWRKY71* can inhibit the GA signal transduction in rice (Zhang et al., 2004). Studies have also demonstrated that *WRKY* transcription factors are critical factors in plant responses involving the ABA signaling pathway, and *WRKY* family members are involved in ABA and IAA signal transduction (Jiang and Yu, 2009; Shang et al., 2010; Rushton et al., 2012). In this study, among the WRKY transcription factors identified, 181 were differentially expressed, and *WRKY32*, *WRKY33* and *WRKY46* were also DMGs. Thus, these transcription factors may regulate flowering-related genes and respond to plant hormone signals, ultimately playing an important role in early flowering.

*LHY*s belong to the MYB transcription factor family, and these proteins positively regulate plant flowering time. In this study, the expression of *LHY* genes in the treatment group was 2.81-fold that of in control group. Both *GmGBP1* and *MYB33* can increase *LFY* expression and promote soybean flowering (Ma et al., 2020). *PtrMYB192* can regulate flowering time by activating the floral inhibitor gene *FLC* (Liu et al., 2013). Plants with mutations in *MYB44* bloomed earlier than wild-type plants did under both long- and short-day conditions, while *MYB44* overexpression resulted in the opposite response (Jaradat et al., 2013). Overexpression of the *AtMYB24* gene can lead to dwarfing, dysplasia of flower organs, lack of anther dehiscence and pollen inactivation (Yang et al., 2007). Many DTFs were also differentially methylated, such as *MYB46*, *MYB35*, and *FLC.*

PIF transcription factors belong to the bHLH family and act directly in photosensitive pigment signal transduction, and PIF4 proteins can directly activate the expression of the *FT* gene (Kumar et al., 2012). Overexpression of the *PpcbHLH1* gene promotes early flowering in *Arabidopsis* (Ren et al., 2016). In this study, most of the bHLH genes were upregulated, such as *PIF1*, *bHLH19*, *bHLH35*, *bHLH36*, *bHLH53*, *bHLH69*, and *bHLH91*. Furthermore, the *PIF1* gene was also differentially methylated gene. Thus, that these genes may play a certain role in the regulation of flowering.

GATA transcription factors also play a key role in the regulation of plant flowering. For example, the GATA transcription factors *GNC* and *GNL* interact with the MADS transcription factor *SOC1* to jointly regulate the flowering time of *Arabidopsis* (Richter et al., 2013). Most GATA transcription factors such as *GATS12* promote flowering (Lu et al., 2017). In the present study, the majority of GATA DTFs was differentially methylated gene, such as *GATA14*, *GATA15*, and *GATA24*. Based on the above research, it was found that 5-azaC pretreatment could regulate the genes of kenaf flowering time by reducing the methylation level of kenaf genome, as well as improving the sugar content and changing the plant hormone content with a systematic and comprehensive research.

## Conclusion

**FIGURE 11:**
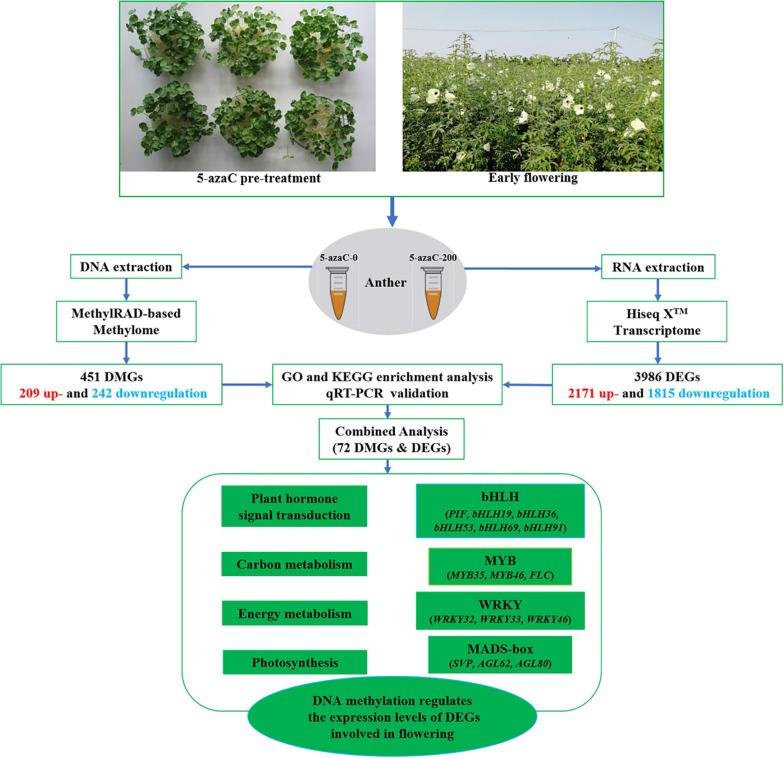
Representative DMGs and DEGs involved in the regulation of flowering.

In this study, pretreatment with the DNA methylation inhibitor 5-azaC induced early flowering in kenaf. 5-azaC could reduce DNA methylation levels in kenaf, resulting in the activation or repression of gene expression. The DMGs, including *ARF*s, *starch synthase*, *FLC*, *PIF1*, *AGL80*, *WRKY32*, and *PP2C*, are closely related to the regulation of flowering time through essential pathways such as plant hormone signal transduction, starch and sucrose metabolism, and the tricarboxylic acid cycle (TCA) cycle (Figure 11). This study is highly important for understanding the potential role of DNA methylation in the regulation of kenaf flowering.

## Data Availability Statement

The datasets presented in this study can be found in online repositories. The names of the repository/repositories and accession number(s) can be found below: Sequence Read Archive (SRA) (https://www.ncbi.nlm.nih.gov/sra/) of NCBI (accession numbers: SRR11743644-SRR11743649 and SRR10912354-SRR10912359).

## Author Contributions

PC conceived the project and revised and edited the manuscript. ZL performed the experiments, analyzed the data, and wrote the manuscript. RL, MT, MK, SC, and YH revised and edited the manuscript. DL, WZ, ZH, and JY assisted in managing the materials. All the authors have read and approved the manuscript.

## Conflict of Interest

The authors declare that the research was conducted in the absence of any commercial or financial relationships that could be construed as a potential conflict of interest.

## Publisher’s Note

All claims expressed in this article are solely those of the authors and do not necessarily represent those of their affiliated organizations, or those of the publisher, the editors and the reviewers. Any product that may be evaluated in this article, or claim that may be made by its manufacturer, is not guaranteed or endorsed by the publisher.

## Footnotes

1
https://ngdc.cncb.ac.cn/gwh/Assembly/21259/show

2
http://samtools.sourceforge.net/mpileup.shtml

3
https://www.ncbi.nlm.nih.gov/sra/

## Supplementary Material

Supplementary Table 1 Sequences of primers used for RT-PCR.

Supplementary Table 2 Distribution of methylation sites.

Supplementary Table 3 Lists of DMGs and DMSs.

Supplementary Table 4 GO and KEGG enrichment of DMGs and DMSs.

Supplementary Table 5 Filtered RNA-seq data.

Supplementary Table 6 Statistics of gene comparison data.

Supplementary Table 7 Genes differentially expressed between 5-azaC200 and 5-azaC0.

Supplementary Table 8 GO and KEGG analysis of DEGs.

Supplementary Table 9 Association analysis of DEGs and DMGs.

Supplementary Table 10 GO and KEGG analysis of DMGs and DEGs.
