# Supplementary material for: Integrated Methylome and Transcriptome Analyses Reveal the Molecular Mechanism by Which DNA Methylation Regulates Kenaf Flowering
Source: Front Plant Sci. 2021 Aug 26;12:709030. doi: 10.3389/fpls.2021.709030 (PMC8428968; doi:10.3389/fpls.2021.709030)
Supplement: Supplementary Table 1 — Sequences of primers used for RT-PCR. [file Table_1.DOCX]

**Table S1** Primer sequences for qRT-PCR.

| Primer | Sequence (5′-3′) | |
| --- | --- | --- |
|  | Forward Reverse | |
| *18SrRNA* (reference gene) | AGAAACGGCTACCACATC | TACTCATTCCAATTACCAGACTC |
| *Actin 3* (reference gene) | GTGAGGATATTCAACCCCTTGTCT | CATCTTTCTGTCCCATACCAACC |
| *Histone3* (reference gene) | GTGGAGTCAAGAAGCCTCACAG | ATGGCTCTGGAAACGCAAA |
| *83B1* | CTTCGTGGTGCTCGCAGATTAT | AGGCGACCAACTTCGTCTTCTC |
| *ABCG25* | GCAAATCTACTCTCCTCAATGCCTT | TCAAGTGAGGGTAGAGAACATCGTC |
| *ABCG26* | GCCTCTCTGGTGTTGATGTTGTTTC | CGCCTTTGCTTTGGCATTCATAC |
| *ARF11* | CGAACAAGAGACCGATGAAGTTTATG | TATCCAGAGGAGGCAGGCACT |
| *CAT6* | CGTCTCGGGACTTTACTGCCTC | TTGCTCCTGCCCCTACAACATT |
| *CKX7* | CTGCCTTGAAGTGGCTTACCATTAC | CGCTTGTTCCTCCGCTCGTT |
| *CSLA2* | CCCCTTACGATTCTGGTTCCC | CGACCCACTCATTGGCTCTG |
| *CSLE1* | AAGGGTTTTCTCAGTGGGATTCA | CGCATCCTTCGCTATCCTTTG |
| *CYP72A219* | TCACTGACCCTGAGTTGATAAGAGAG | GCTTAGCCCACTCATCTCCCTC |
| *Egs* | GCGGCTCGGTCCATTTCT | CTTGCTCGGGTGGCTGTG |
| *GSO1* | ATGCCGAGGCTGCTTGG | GAACTGAGCAAACAGGGTGAGG |
| *IAAldO* | AGCGGAAATCATAAAGACCGTGTT | ACCTGCCGTCTGGCTTCTCTTAC |
| *IRX9* | CATTGAAGCACATTGAGCAGC | TTCTCTTGTTTGCTGATAGTAATGC |
| *laccase-22* | GTTCCTAACTGTTCTTCTGATGATGC | GAAGAGTTCATCATTCACAGCCG |
| *LIN* | AGTCCTTCGGATTCCACGGT | TCCTCTCGGAACACACTTTTACC |
| *MAPK7* | TTGTATTGCCCCCCTAACCGT | TGGAATCTCGTTGAAGTTCTAAGGC |
| *PP2C46* | ACAATGTGTGGCGTGTAAAAGGTAT | GAACTACGGCTAAACCAAACTCCC |
| *PP2C60* | GAGTTCTCAATGGCAGTGGTTCAGG | CCTTGTTGCTCTGAAGTGAATCGTTT |
| *PPR* | CGTGAGGAAGCATTGCCAGAG | CACTAAATCCTCCCTTGTTGCCAC |
| *rps5* | CAGTTCGGATTGTGAAGCATACC | CTGATTCACACGACGAAGAGGAG |
| *SIK-CDL1* | AGGGGAGTTCTTAGTAATGGCAGG | CTATCCGAGCAATAACCAATCAAAG |
| *SLC2* | TCACGGTATTCTTTTGTGGGATTGT | GCCATAACAAGACCTATTAGCACAGC |
| *ZC3H6* | TAGCCGAATCCCTATTGTTCCTCT | CGGAGTTCCAGAGTTTGACAGACC |
| *β-1,2XylT* | AGACCCCACTTACCAACGCCG | GCAGCGAAACCAACTGGAGCC |
