# Supplementary material for: Integrated Methylome and Transcriptome Analyses Reveal the Molecular Mechanism by Which DNA Methylation Regulates Kenaf Flowering
Source: Front Plant Sci. 2021 Aug 26;12:709030. doi: 10.3389/fpls.2021.709030 (PMC8428968; doi:10.3389/fpls.2021.709030)
Supplement: Supplementary Table 5 — Filtered RNA-seq data. [file Table_5.docx]

**Table S5** Filtered date of RNA-seq.

| Sample | Raw-reads | Raw-bases | Clean-reads | Clean-bases | Valid-bases | Q30 | GC |
| --- | --- | --- | --- | --- | --- | --- | --- |
| 5-azaC0-1 | 49434578 | 7415186700 | 47661372 | 6847814878 | 92.35% | 94.12% | 46.25% |
| 5-azaC0-2 | 48988754 | 7348313100 | 47536320 | 6844820648 | 93.15% | 94.27% | 46.27% |
| 5-azaC0-3 | 49730874 | 7459631100 | 48442546 | 6990053865 | 93.71% | 94.49% | 46.13% |
| 5-azaC0 (average) | 49384735 | 7407710300 | 47880079 | 6894229797 | 93.07% | 94.29% | 46.22% |
| 5-azaC200-1 | 49366982 | 7405047300 | 48177668 | 6949374904 | 93.85% | 94.72% | 46.04% |
| 5-azaC200-2 | 49897380 | 7484607000 | 48711946 | 7041783660 | 94.08% | 94.70% | 46.07% |
| 5-azaC200-3 | 49114892 | 7367233800 | 47853740 | 6906009126 | 93.74% | 94.67% | 46.17% |
| 5-azaC200 (average) | 49459751 | 7418962700 | 48247785 | 6965722563 | 93.89% | 94.70% | 46.09% |
